# Fine Needle Aspiration

**DOI:** 10.5812/traumamon.16848

**Published:** 2014-01-25

**Authors:** Viroj Wiwanitkit

**Affiliations:** 1 Department of Tropical Medicine, Hainan Medical University, Haikou, China

**Keywords:** Diagnosis, Biopsy, Fine-Needle, Sensitivity and Specificity, Predictive Value of Tests

*Dear Editor,*

The recent report on fine needle aspiration was very interesting. Akhavan-Moghadam noted that “fine needle aspiration (FNA) is a useful atraumatic diagnostic technique with high diagnostic accuracy, which can provide a highly sensitive and low false positive diagnosis in patients with nonthyroidal masses” (1). It is interesting to discuss the diagnostic properties of FNA and its harmfulness. According to a recent study by Wharry et al., it was reported that the false negative results were as high as 10.4 %, which may cause problems in diagnostic process (2). The needle size and experience of cytologist are the main factors that determine the false results (3). Furthermore, although FNA is classified as a minimally invasive technique, there are still some serious complications such as cyst fluid leakage, pneumothorax, anaphylactic reaction and thromboembolism that should be considered by all practitioners performing FNA (4).
